# Autoimmune glial fibrillary acidic protein astrocytopathy associated with breast cancer: a case report

**DOI:** 10.1186/s12883-023-03194-7

**Published:** 2023-04-04

**Authors:** Tomonori Yaguchi, Akio Kimura, Akira Takekoshi, Mikiko Matsuo, Hiroyuki Tomita, Takayoshi Shimohata

**Affiliations:** 1 Department of Neurology, Gifu Prefectural General Medical Center, Gifu, Japan; 2 Department of Neurology, Gifu University Graduate School of Medicine, 1-1 Yanagido, Gifu, 501-1194 Japan; 3 Department of Tumor Pathology, Gifu University Graduate School of Medicine, Gifu, Japan

**Keywords:** Astrocytopathy, Autoantibody, Breast cancer, Glial fibrillary acidic protein (GFAP)

## Abstract

**Background:**

Autoimmune glial fibrillary acidic protein (GFAP) astrocytopathy (GFAP-A) is an autoimmune inflammatory central nervous system disorder characterized by the detection of autoantibodies that recognize GFAP in CSF. The pathogenesis of GFAP-A is poorly understood. Some patients had a neoplasm detected and GFAP expressed by neoplasms is plausible as immunogen triggering paraneoplastic neurological autoimmunity.

**Case presentation:**

We report a case of 76-year-old female patient with GFAP-A complicated with breast cancer. She presented with altered consciousness, nuchal rigidity, speech disturbances, and weakness. Her clinical symptoms were improved by immunotherapy and cancer treatments. Immunohistochemical analysis showed that the restricted tumor expressed GFAP. The infiltration of CD3 + T cells were observed in the peritumoral and intratumoral areas. The most common infiltrating lymphocytes were CD8 + T cells. CD4 + T cells and CD20 + B cells were also observed in the predominant peritumoral area.

**Conclusions:**

These results suggest that GFAP-A may occur in a paraneoplastic neurological syndrome associated with breast cancer.

## Introduction

Autoimmune glial fibrillary acidic protein (GFAP) astrocytopathy (GFAP-A) was first reported as an autoimmune inflammatory central nervous system disorder characterized by the detection of immunoglobulin G (IgG) antibodies that recognize GFAP, which is the main intermediate filament protein in mature astrocytes [1]. Further studies confirmed that tissue- and cell-based testing, using cerebrospinal fluid (CSF), determined the highest specificity for inflammatory central nervous system (CNS) diseases [2, 3]. In some GFAP-A patients, GFAP-IgG is only detected in CSF, while patients with GFAP-IgG in serum only (not in CSF) have diverse neurological phenotypes that may or may not have an autoimmune cause. The use of one assay alone may yield nonspecific results. The sensitivity and specificity of the “GFAP pattern” in tissue-based testing for predicting GFAP-IgG were previously reported as 95.2% (95% CI 76.2–99.9) and 90.0% (95% CI 55.5–99.8), respectively [4]. At present, the diagnostic criteria for GFAP-A have not been established and detection of CSF GFAP-IgG by tissue- and cell-based testing is essential for a diagnosis of GFAP-A [2, 3]. In recent years, the clinical features of GFAP-A have become better understood [2, 3, 5–9]. The common phenotype of this disorder includes meningoencephalitis with or without myelitis, and the common prodromal symptoms are fever and headache. During the clinical course, patients present with consciousness disturbances, meningeal irritation, ataxia, involuntary movements such as tremor and myoclonus, urinary dysfunction, cognitive dysfunction, and respiratory failure. Blurred vision related to optic disc edema is also observed [8, 9]. A recent French GFAP-A cohort study found the median age at onset to be 43 years, and that 65% of patients were men. Infectious prodromal symptoms were found in 82% of patients. The most frequent presentation was subacute meningoencephalitis (85%), while cerebellar dysfunction was observed in 57% of cases [9]. There is currently no consensus on treatment regimens for this disorder. The response to corticosteroid therapy is generally good. Sometimes intravenous immunoglobulin therapy or plasma exchange may be performed in addition to corticosteroid therapy. However, some patients experience relapses with bad prognoses, including death. Treatments for refractory or relapsing cases include mycophenolate mofetil, azathioprine, rituximab, cyclophosphamide, and tacrolimus [9, 10].

Cerebrospinal fluid (CSF) can be probed for lymphocyte-predominant pleocytosis and elevated protein levels. Brain magnetic resonance imaging (MRI) can show abnormal hyperintensity lesions on T2-weighted and fluid-attenuated inversion recovery (FLAIR) images [8]. Brain linear perivascular radial gadolinium-enhancement (LPRGE) patterns, an imaging hallmark of GFAP-A, are observed in about half of all patients [2, 8].

The pathogenesis of GFAP-A is poorly understood. Pathologically, there is a marked lymphocytic infiltration of the brain parenchyma, with many CD8 + and CD4 + T cells, especially in the perivascular area [11–13]. GFAP-specific cytotoxic T cells are also likely effectors of this disorder [2, 14]. Approximately 20% of patients have a neoplasm [14]. GFAP expressed by neoplasia is plausible as an immunogen-triggering paraneoplastic neurological autoimmunity. The most commonly detected neoplasia is ovarian teratoma [2]. Other neoplasms are rare and diverse. Herein, we report the clinical features and the pathological findings of tumor tissue in a GFAP-A patient with breast cancer.

## Case report

A 76-year-old woman who had a past medical history of hypertension, atrial fibrillation, and right thalamic hemorrhage was admitted to the hospital because of fever and impaired balance. She suffered neck pain and dysarthria two days after admission and became somnolent. CSF examination showed pleocytosis (130 cells/mm^3^ [mononuclear cell: 130 cells/mm^3^], normal < 5 cells/mm^3^) and increased protein levels (113 mg/dL, normal < 50 mg/dL). She was treated with intravenous acyclovir on suspicion of viral meningoencephalitis. Her symptoms did not improve, and she was referred to our hospital for further investigation and treatment 22 days after onset. On admission, she was afebrile, and her consciousness level was E3V3M5 on the Glasgow Coma Scale. Her speech was slurred and barely comprehensible. She had left hemiparesis because of a past thalamic hemorrhage and flaccid muscle weakness in her right lower extremity. Tendon reflexes were hyperreflexia in both upper limbs and areflexia in both lower limbs. She had nuchal rigidity, but Kernig’s sign and Brudzinski’s sign were not observed.

Peripheral blood cell counts showed mild thrombocytopenia (141 × 10^3^ /µL, normal range: 158–348 × 10^3^/µL). Biochemical examinations showed hypoalbuminemia (2.8 g/dL, normal range: 4.1–5.1 g/dL) and an elevated urea-nitrogen creatinine ratio suggestive of dehydration. C-reactive protein was within the normal limit. Serum thyroid stimulating hormone and free thyroxine levels were within normal limits. A mild elevated carbohydrate antigen 19 − 9 level was observed (39.2 U/mL, normal ≤ 37.0 U/mL). Serum anti-nuclear antibody and anti-aquaporin 4 antibody were negative. Anti-neuronal antibodies including anti-amphiphysin, CV2, Ma2, Ri, Yo, Hu, recoverin, SRY-related HMG-box gene 1, titin, zinc-finger protein of the cerebellum 4, Tr, and glutamic acid decarboxylase 65 antibodies were all negative results (Euroimmun, Lübeck, Germany). Mycobacterium tuberculosis specific interferon-gamma release assay and serum *Candida*, *Aspergillus*, and *Cryptococcus* antigens were negative.

**Fig. 1:**
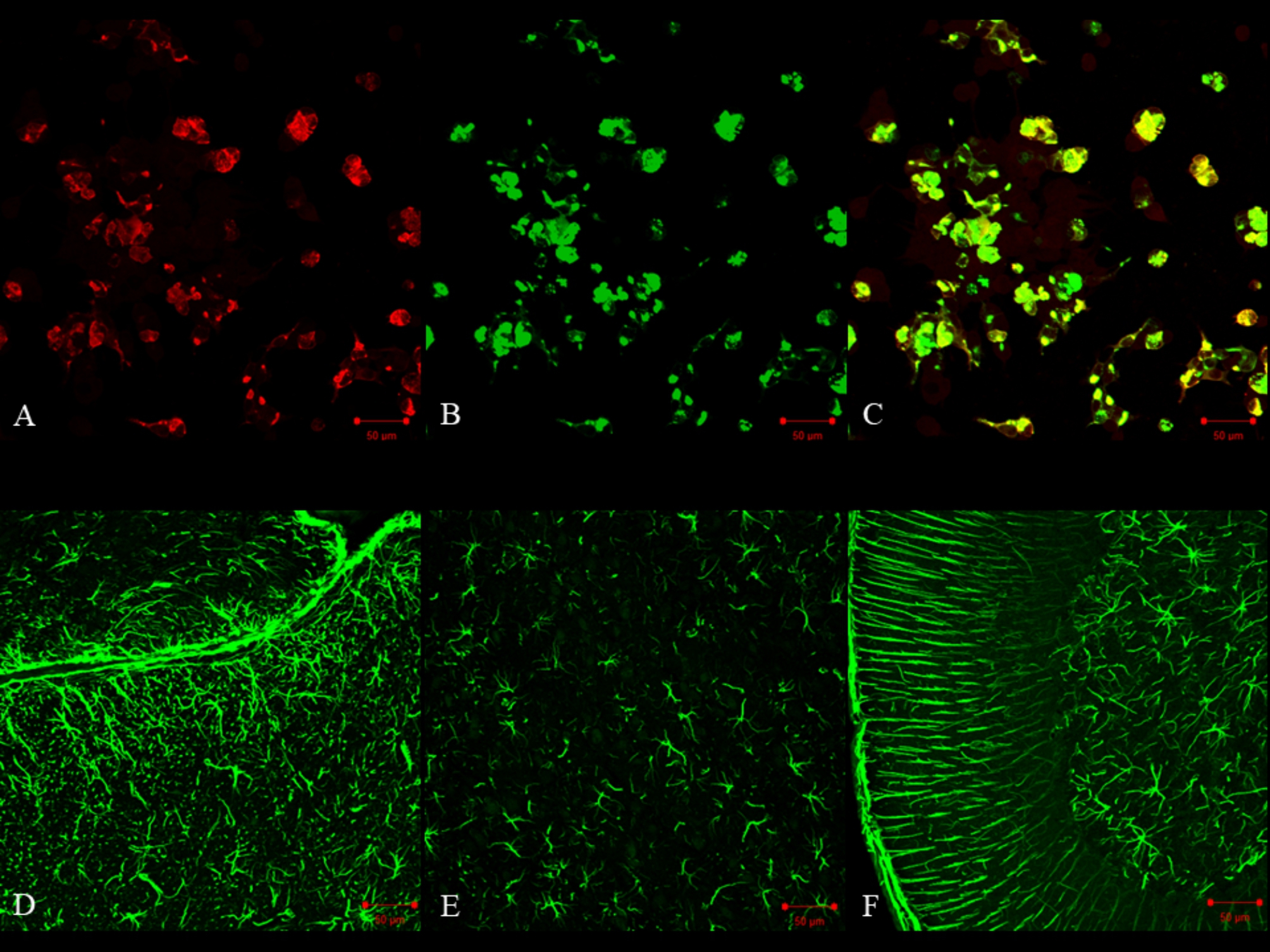
Detection of cerebrospinal fluid (CSF) glial fibrillary acidic protein (GFAP) immunoglobulin G (IgG). Cell-based assay of GFAPα-transfected HEK293 cells (AC). GFAP-IgG was detected in the CSF of the patient with autoimmune GFAP astrocytopathy (A). HEK293 cells stably express green fluorescent protein (GFP)-tagged GFAPα (B). Colocalization of the patient’s CSF-IgG and GFAPα is yellow in merged images (C) Tissue-based immunofluorescence assay (D–F). Immunoreactivity of the patient’s CSF-IgG was observed in astrocytes of the pial, subpial (D), parenchyma (E), and cerebellum (F)

CSF examination showed normal opening pressure (105 mmH_2_O), pleocytosis (42 cells/mm^3^ [mononuclear cell: 41 cells/mm^3^]), increased protein levels (95 mg/dL), and mildly decreased glucose levels (40 mg/dL). Bacterial culture had a negative result. Herpes simplex virus and *Mycobacterium tuberculosis* polymerase chain reaction tests also gave negative results. CSF cytology showed no malignant cells. Later, CSF GFAP-IgG was detected by transfected cell-based assay and tissue-based immunofluorescence assay according to previous reports [2, 5] (Fig. 1).

**Fig. 2:**
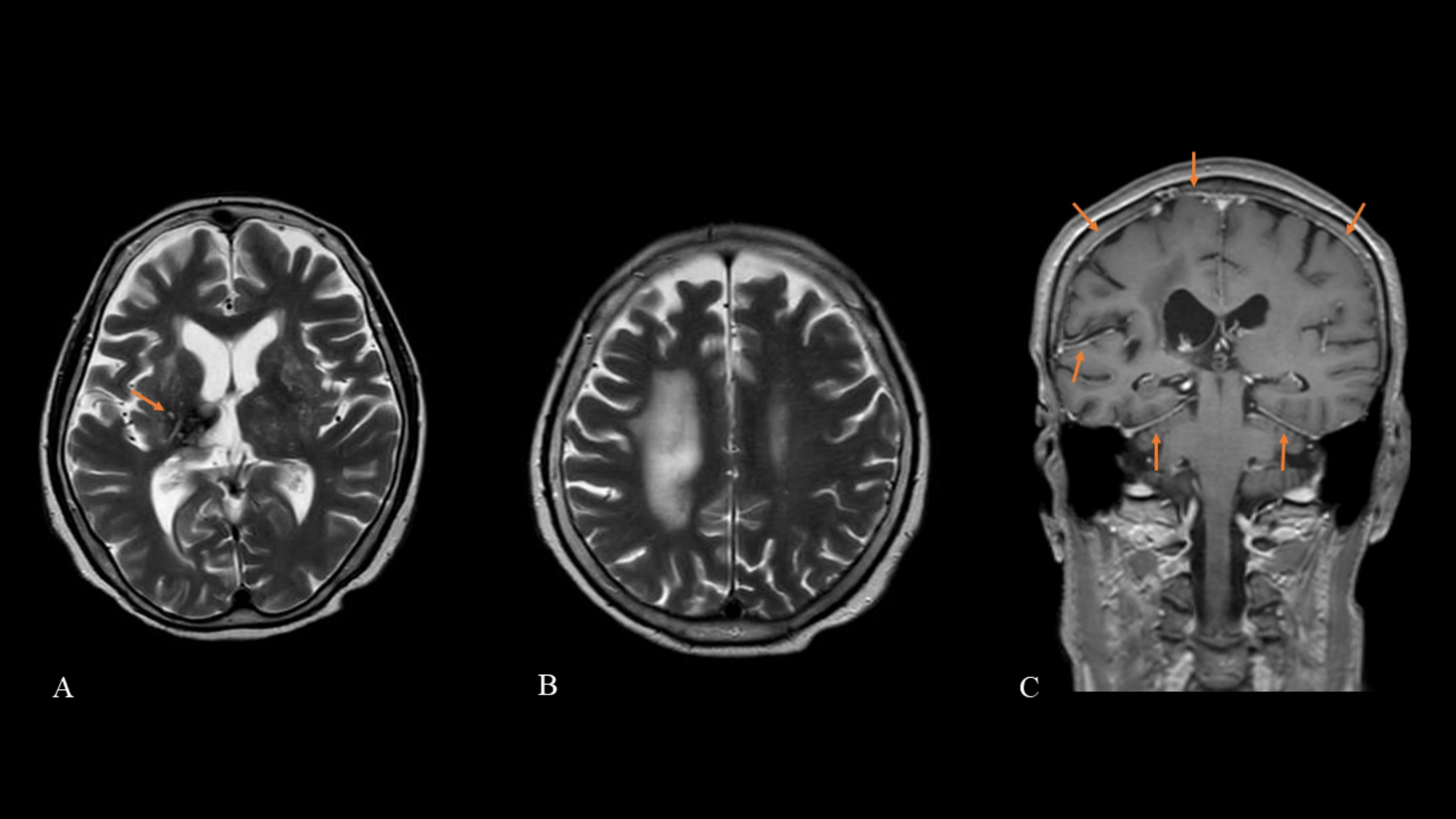
Brain magnetic resonance imaging Brain magnetic resonance T2-weighted images show abnormal signal changes caused by a past right thalamic hemorrhage (arrow) (A) and extended white matter hyperintensity lesions in the deep and periventricular white matter in the right frontal and parietal lobes (B). Gadolinium contrast-enhanced brain MRI scans show heterogeneous thickening of the dura mater (arrows) (C)

Brain MRI scans showed abnormal signal changes caused by a past right thalamic hemorrhage (Fig. 2A) and extended white matter hyperintensity lesions in the deep and periventricular white matter in the right frontal and parietal lobes on T2-weighted and FLAIR images (Fig. 2B). Gadolinium contrast-enhanced brain MRI scans showed heterogeneous thickening of the dura mater (Fig. 2C). Spinal MRI showed no abnormal signal changes in the spinal cord. Whole body computed tomography (CT) showed no findings of neoplasia. However, early-stage breast cancer was found in the left breast on mammography.

Based on these results, the patient was diagnosed with GFAP-A. She was treated with an intravenous infusion of 1 gram per day methylprednisolone for 3 days starting on day 8 after admission. Her nuchal rigidity disappeared on day 10. Her consciousness level gradually improved starting on day 11 and became completely clear on day 32. She was also treated with intravenous immunoglobulin (0.4 gram per kilogram body weight for 5 days) on day 36 and again on day 60. She was temporarily transferred to the local hospital on day 91 for rehabilitation.

**Fig. 3:**
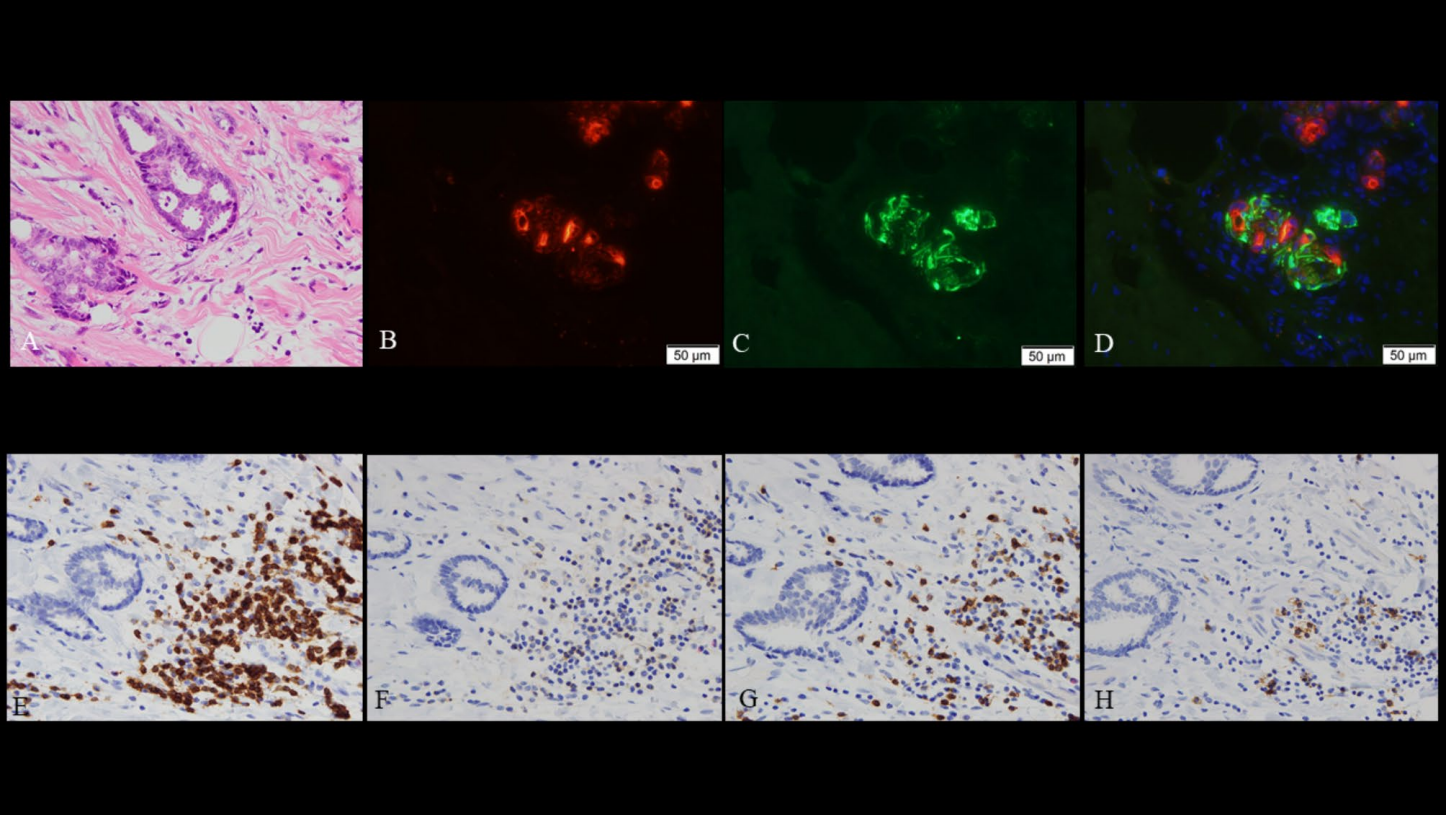
Breast cancer pathology Hematoxylin-Eosin (HE) staining (A) and immunohistochemical analysis using commercial antibodies against pan-cytokeratin AE1/AE3 (B) show invasive ductal carcinoma. Immunohistochemical analysis using commercial antibodies against GFAP (C) and the merged image of staining AE1/AE3 and GFAP (D) reveals the expression of GFAP in breast tumor tissue. Immunohistochemical analysis shows that the infiltration of CD3 + T cells were observed in peritumoral and intratumoral areas (E). The most common infiltrating lymphocytes were CD8 + T cells (F). CD4 + T cells (G) and CD20 + B cells (H) were also observed in predominant peritumoral areas

A month later, the patient was re-admitted to our hospital and underwent a simple mastectomy for breast cancer. The pathological findings were invasive ductal carcinoma, tubule forming type (Fig. 3A, B). Immunohistochemical analysis showed that the restricted tumor expressed GFAP (Fig. 3C, D). The infiltration of CD3 + T cells were observed in the peritumoral and intratumoral areas (Fig. 3E). The most common infiltrating lymphocytes were CD8 + T cells (Fig. 3F). CD4 + T cells and CD20 + B cells were also observed in the predominant peritumoral area (Fig. 3G, H). Her condition did not deteriorate, and no relapse occurred thereafter.

## Discussion

Here, we provide a report on a GFAP-A patient with breast cancer. This patient was initially suspected to have viral meningoencephalitis and treated with an antiviral therapy, but her symptoms did not improve. Afterwards, she was diagnosed with GFAP-A, and her condition improved after receiving immunotherapy and cancer treatments.

Previously, Flanagan et al. reported that diverse neoplasms were detected in 22 of 102 patients subsequent to neurological presentation [2]. The most commonly detected neoplasia in these cases was ovarian teratoma. Another previous report described that an ovarian teratoma from a patient with GFAP-A with coexisting N-Methyl-D-Aspartate Receptor (NMDAR)-IgG had robust GFAP staining and sparse NMDAR staining [5]. Another group also reported that an ovarian teratoma associated with coexisting NMDAR and GFAP autoimmune meningoencephalitis in an adolescent girl had extensive CD3 + T cell infiltration [16]. In contrast, many other types of neoplasms have been reported in this condition, including adenocarcinoma (endometrium, esophagus, kidney, prostate, colon, ovary, lung, breast), gliomas, multiple myeloma, pleomorphic parotid adenoma, carcinoid, squamous cell carcinoma (head, neck, nasopharyngeal), small cell lung carcinoma, Hodgkin lymphoma, B cell lymphoma, chronic lymphocytic leukemia, melanoma, renal cell carcinoma, breast ductal carcinoma, urothelial bladder carcinoma, meningioma, and thymoma [2–6, 17]. The true association between these neoplasms and GFAP-A has not been clarified. However, in this study, we have provided the first identification of GFAP expression in breast cancer tumor tissue and the extensive infiltration of CD3 + T cells, including CD8 + T cells, CD4 + T cells, and CD20 + B cells in peritumoral and intratumoral areas. Although we have not confirmed that GFAP is absent in breast cancer cells without GFAP-A, database searches (https://www.proteinatlas.org) show that GFAP is not expressed in breast cancer cell lines [18]. We suggest that the breast cancer is an immunogen that triggers GFAP-A. When GFAP-A is diagnosed in adult females, mammary glands should be included in screening for neoplasms. In addition, meningoencephalitis, with or without myelitis, in patients with breast cancer may be due to this syndrome, and therefore, the CSF levels of GFAP-IgG should be examined. Although the pathophysiological mechanisms of GFAP-A remain to be elucidated, GFAP-specific CD8 + T cells are likely effectors of this disorder [2]. It is possible that ectopic expression of GFAP in the neoplasm triggered the autoimmune response against GFAP including the production of GFAP-specific CD8 + T cells and B cells. After disruption of the blood–brain barrier by infection, these cells can infiltrate the CNS and cause GFAP-specific CD8 + T cell-related inflammation and GFAP-IgG production.

In the patient described in this study, gadolinium contrast-enhanced brain MRI scans showed a heterogeneous thickening of the dura mater. Similarly, a recent report described a pediatric patient with hypertrophic pachymeningitis associated with GFAP-A, and the authors indicate that hypertrophic pachymeningitis may be one of the clinical phenotypes for GFAP-A [19], which is also corroborated by our findings.

The limitations of this study are as follows. First, this study is a case report and lacks information on other potential risk factors for GFAP-A. To further examine the association between breast cancer and GFAP-A, it will be necessary to identify additional GFAP-A patients who also present with breast cancer. Second, differences in inflammatory cells infiltrating tumor tissues of breast cancers with and without GFAP-A are not known. Differences between these tumor tissues and the presence of GFAP antigen-specific lymphocytes in tumor tissue with GFAP-A warrant further investigation.

## Conclusion

Here, we report a patient with GFAP astrocytopathy, which may occur in a paraneoplastic neurological syndrome associated with breast cancer.

## Data Availability

All data reported within the article are available as an anonymized set by request from qualified investigators.

## Abbreviations

CSF: cerebrospinal fluid
CT: computed tomography
FLAIR: fluid-attenuated inversion recovery
GFAP: glial fibrillary acidic protein
GFAP-A: Autoimmune glial fibrillary acidic protein astrocytopathy
IgG: immunoglobulin G
LPRGE: linear perivascular radial gadolinium-enhancement
MRI: magnetic resonance imaging
NMDAR: N-Methyl-D-Aspartate Receptor
